# Quarterly Report of the Obstetric Practice in the Philadelphia Dispensary, Fourth, Fifth and Sixth Months, 1839

**Published:** 1839-07-20

**Authors:** Joseph Warrington

**Affiliations:** Accouchuer


MEDICAL EXAMINER.

DEVOTED TO MEDICINE, SURGERY, AND THE COLLATERAL SCIENCES.

No. 29.] PHILADELPHIA, SATURDAY, JULY 20, 1839. [Vol. II.

*Quarterly Report of the Obstetric Practice in the Philadelphia Dispensary, Fourth, Fifth and Sixth months*, 1839. By Joseph Warrington, M. D., Accouchuer.

Seventeen women have been delivered at full time, one at about 8 months, and one at about 4½ months of gestation, making 19 cases which have been under the care of the institution since last report.

Ten boys and nine girls have been born during this time, one woman having twin boys. The sex of the abortion was not noted. In twelve cases, in which the position of the fœtus was carefully observed, four presented in the first, six in the second, and two in the fourth position of the vertex. The twins presented, the first in the fourth, and the second in the second position.

The average duration of labour in twelve cases was four hours and twenty minutes, the extremes being two and twelve hours.

The average time required for the spontaneous expulsion of the placenta in eight cases was 16 minutes, the extremes being two and thirty minutes.

In two other cases it was necessary to introduce the hand partially into the uterus; in one of them for the purpose of dilating the os uteri which had contracted upon the placenta; and in the other for the separation of a portion of the placenta and membranes which were adherent to the parieties of the uterus. The placenta when removed, was found studded with numerous points of ossification. The child, however, was well developed, and the mother recovered without accident.

The subject of labour at eight months was in the last stages of pulmonary consumption, and in a state of extreme prostration when contractions of the uterus came on and expelled the child without any marked effort on the part of the mother, who died eight days after; the lacteal secretion not having taken place.

One patient who had a very easy labour was attacked with metritis the third day after delivery. The affection was promptly removed by free, repeated bleeding from the arm, saline cathartics, fomentations to the hypogastrium and mucilaginous injections into the vagina. She was convalescent on the 5th day after the attack.

The children have all done well.

Dr. Boyer, of the North Middle District, states that while attending M. H., he was consulted by his wife, a tall, large woman, of sanguine temperament, who described herself as being seven months pregnant. She had at that time a vaginal hæmorrhage of several days duration. The discharge, she said, was fluid, and persisted in stating it at a pint per diem. It was attended with pain, increased by exertion, headach, flushed countenance, full pulse, but no decided heat of skin. She had had more or less hæmorrhage in all her previous pregnancies, and also at an earlier period of this,—had not had a living child for six years, but had aborted several times at an early stage of gestation, during the three years succeeding the birth of her last child. She was vague in the account of her labours, but recollected that she had once been delivered by turning. Subsequently to her last abortion, she was treated for some uterine affection, by leeches and cauterization, and had never regained her health, though she menstruated with regularity for some time previously to her present pregnancy. Directed vss., opiates and astringents in combination, and rest. Rest, owing to circumstances, was only observed when the copiousness of the discharge created alarm, and then never more than 12 hours at a time. The hæmorrhage continued variably for two weeks—seldom abundant as beforementioned, and sometimes absent for a whole day, and finally subsided. Thinking herself in labour, a week or more afterwards I was sent for, and found the os uteri undeveloped, the lips thicker and firmer than natural, and a nipple-like prominency on one lip, while the other gave to the finger the sensation of a depression, with ragged edges and a surface wanting in natural smoothness. The same characters were recognised at a subsequent examination, on a like occasion, early in June. She was delivered by the natural efforts of a large child. The labour was in nothing remarkable, though somewhat prolonged.

This case was not one of those very rare ones of menstruation during pregnancy, since it occurred at only two periods, and was then continued too long for that function. It is probable, though not demonstrable, that it was not from the placenta, but was from an ulcer on the lip of the os uteri. The mother seemed benefited by the loss of blood, and the size of the child proved that it was not injured by it.

